# Interaction between number of formed bubbles, reaction rate and flow velocity of acoustic streaming in the initiation period of power-induced quenching

**DOI:** 10.1016/j.ultsonch.2026.107898

**Published:** 2026-05-22

**Authors:** Ryota Aoki, Takuya Yamamoto

**Affiliations:** Department of Chemical Engineering, Graduate School of Engineering, Osaka Metropolitan University, 1-1, Gakuen-cho, Naka-ku, Sakai, Osaka 599-8531, Japan

**Keywords:** Sonochemical reaction, Power-induced quenching, Rectified diffusion, Sono-chemical luminescence (SCL), Acoustic cavitation, Acoustic streaming

## Abstract

To elucidate the detailed process of power-induced quenching in which the rate of sonochemical reactions decreases sharply as ultrasonic power increases, the interaction between the number of formed bubbles, the sonochemical reaction rate, and the flow velocity of acoustic streaming was investigated during the initiation period of ultrasonic irradiation. The investigation is conducted through the observation of formed bubbles, particle image velocimetry (PIV) measurement, and sono-chemical luminescence (SCL) observation. The experimental findings suggest that the SCL intensity decreased after irradiation for a certain period at the ultrasonic power at which the power-induced quenching occurred. During the quenching process, the number of formed bubbles initially decreased due to the waveform distortion caused by sound waves emitted during the oscillations of the bubbles. Subsequent to the decline in the number of bubbles, the SCL intensity and the flow velocity of acoustic streaming decreased. As the ultrasonic power increases, the decrease in time of SCL intensity, number of formed bubbles, and the flow velocity of acoustic streaming becomes shorter. These phenomena can be interpreted by rectified diffusion, the mass flux of which is largely varied due to the ultrasonic wave distortion.

## 1 Introduction

An acoustic cavitation occurs when a high-intensity ultrasound is irradiated into a liquid [1], [2]. The cavitation bubble is oscillated in accordance with the imposed ultrasound wave. The phase between the bubble and ultrasound oscillations is shifted due to the inertia of liquid motion around the bubble in the high-pressure amplitude zone. This phase shift causes violent bubble collapse, a phenomenon referred to as Rayleigh collapse. The interior of the bubble undergoes a semi-adiabatic process during the collapse period because the collapse time is very short [3], [4]. During this collapse phase, the inner temperature and pressure exceed 5000 K and 100 atmospheric pressure, respectively, thereby inducing thermal decomposition of molecules within the bubble, leading to the formation of highly reactive radicals [5]. The radicals formed in the bubble react with chemical species near the gas-liquid interface, and many chemical reactions proceed simultaneously [5]. This chemical reaction is called sonochemical reaction. In addition to the chemical reaction, micro-jets are formed during the bubble collapse in the zone where the bubble collapse becomes asymmetric e.g. near a wall [6], [7]. Acoustic streaming is developed due to the attenuation of ultrasound [8], [9]. Consequently, many physical and chemical phenomena occur simultaneously, making them difficult to be understood. A series of these phenomena can be used for many applications including, but not limited to, particle dispersion [10], [11], emulsification [12], [13], atomization [14], [15], extraction [16], [17] and organic chemical decomposition [18], [19], [20].

In these applications, the control of sonochemical reactions is of significantly importance for stable process operation, though the precise control of these reactions is difficult. For instance, the acoustic shielding effect narrows the sonochemical reaction zone [21], [22], and the reaction rate is changed by a fluid flow in a sono-reactor [23], [24], [25], concentration of a dissolved gas [26], [27], liquid temperature [28], and etc. Moreover, the sonochemical reactions are largely reduced by excessive ultrasonic power [29], [30], [31], [32], [33], [34], [35], [36], addition of alcohol [37], [38], [39], [40], [41], and surfactants [42], [43]. This phenomenon is called quenching of sonochemical reaction. A variety of phenomena have been interpreted in terms of followings: the bubble-bubble interactions, adsorption of solute and/or surfactant along the bubble interface, coalescence of bubbles, clustering of bubbles, suppression of bubbles, and nonlinear wave distortion. Because all the above factors influencing the quenching of sonochemical reactions have interacted each other, the detailed process of quenching have been still unclear. In order to elucidate the detailed process of power-induced quenching, an investigation was conducted into the interaction between the quenching of sonochemical reactions, ultrasonic wave characteristics, fluid velocity of acoustic streaming, number of formed bubbles, stability of acoustic bubbles, and oscillation characteristics of cavitation bubbles [36], [44]. In this previous study, the following process of power-induced quenching was proposed.

1. An ultrasonic wave is distorted by the emission of sound wave from the bubble oscillations. This distortion in the ultrasonic wave results in alterations to the bubble oscillations, reducing the mass transfer of dissolved gas due to rectified diffusion, and consequently, the number of formed bubbles decreases.
2. The decrease in the number of active bubbles leads to a reduction in the sonochemical reaction rate throughout the entire vessel.
3. The decrease in the number of active bubbles reduces the sound attenuation, leading to the decrease in the velocity of acoustic streaming.
4. The decrease in the number of active bubbles reduces the degassing rate due to the diminished rectified diffusion.

Despite the fact that this proposed mechanism is based on the six experimental results, numerical simulation and stability analysis, the interaction between the bubble growth rate, sonochemical reaction rate, and velocity of acoustic streaming has not been explained solely from the experimental evidence. Therefore, this study attempts to experimentally elucidate the relationship between bubble generation rate, quenching, and acoustic flow velocity reduction. To elucidate this phenomenon, the initial stage of ultrasonic irradiation was examined because the suppressions of formed bubbles, acoustic streaming, and sonochemical reactions can be measured at this stage. The relationship between the macroscopic bubble generation rate, changes in reaction rate, and changes in flow velocity of acoustic streaming during this initial irradiation period was elucidated through meticulous measurement.

## 2 Experiment

**Fig. 1:**
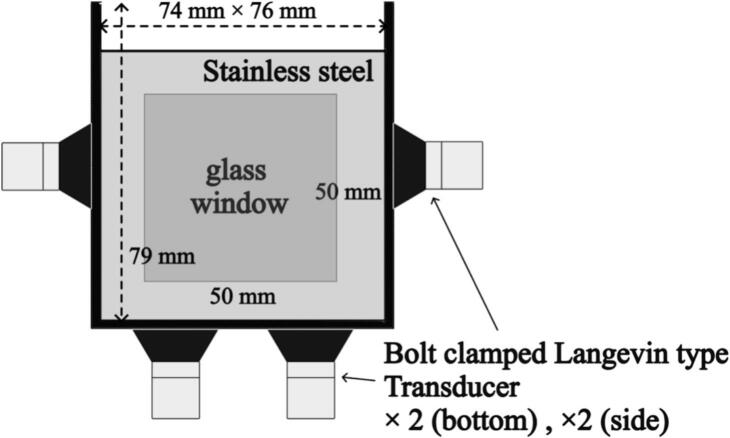
Schematic drawing of experimental setup.

The experimental setup involved the utilization of an ultrasonic bath as depicted in Fig. 1, and the operating frequency is 154 kHz. The experimental setup employed in this study is the same as that used in our previous study [36]. The vessel is rectangular in shape and is equipped with 50 mm × 50 mm window frames made of glass, allowing us to observe the insides of the vessel. The ultrasonic bath, with dimensions measuring 74mm × 76 mm × 79 mm, contains four bolt clamped Langevin type transducers affixed to the vessel walls as shown in Fig. 1. The vessel wall was composed of stainless steel. For all experiments, 400 mL aqueous solution was used, and to minimize the free surface oscillations, a square acrylic plate was placed on the free surface. The effective electric power, namely ultrasonic power *P*_U_ was used to characterize the power input in the aqueous solution. Due to differences in acoustic impedance, incident ultrasonic waves primarily propagate within the ultrasonic bath. Most of the ultrasonic energy is absorbed by the water and converted into thermal energy. Therefore, measuring the temperature increase in an insulated ultrasonic tank allows one to determine the ultrasonic power incident on the water, *P*_U_ and this method is called calorimetry method. The ultrasonic bath was covered by a glass wool insulator, and a thermocouple (Okazaki Manufacturing company, AEROPAK) was inserted into the solution. The temperature increase during ultrasonic irradiation was measured in this system for each electric power, and the temperature gradient over time was calculated especially in the initial period of ultrasonic irradiation. Finally, the ultrasonic power was calculated from the temperature gradient over time using the following equation.

$$\begin{array}{c} P_{U}=MC_{p}\frac{\mathrm{dT}}{\mathrm{dt}} \end{array}$$

where, *M* is the mass of pure water, *C*_p_ is the heat capacity, *T* is temperature measured by the thermocouple, and *t* is time. To evaluate the effect of power input on the sonochemical reactivity, the electric power was varied at 30, 50, 80, 125, 150, 175, and 200 W. It means that the ultrasonic power, *P*_U_ was varied at 18, 33, 43, 69, 84, 95, and 107 W. This power input corresponds to the pressure amplitude at the irradiation surface, *P* of approximately 0.4 to 1.1 atmospheric pressure, which is calculated by the following equation.

$$\begin{array}{c} P=\sqrt{\frac{\rho_{L}cP_{U}}{A}} \end{array}$$

where *ρ*_L_ is the liquid density, *c* is the speed of sound in liquid, and *A* is the irradiation area. The parameters and used physical properties for Eqs. (1) and (2) are summarized in Table 1.

**Table 1:** Parameters and physical properties used in this study.

Variable	Value	Unit
Mass of pure water, *M*	3.99 × 10^-1^	kg
Heat capacity, *C*_p_	4.18 × 10^3^	J/(kgK)
Liquid density, *ρ*_L_	9.98 × 10^2^	kg/m^3^
Speed of sound in liquid, *c*	1.48 × 10^3^	m/s
Irradiation area, *A*	1.63 × 10^-2^	m^2^

### 2.1 Sono-chemical luminescence (SCL)

The chemical reaction rate and reaction zone were measured by sono-chemical luminescence (SCL) in the luminol aqueous solution. The mass concentration of luminol was 0.01 wt%. Additionally, Na_2_CO_3_ was added to elevate pH of the aqueous solution to an alkaline level, which is required for dissolving luminol in a water. The concentration of Na_2_CO_3_ was 0.5 wt%. Luminol emits blue light when exposed to hydroxyl radicals and other oxidants during ultrasonic irradiation. The blue light was recorded by an electron multiplying CCD camera (Andor, iXon Ultra, DC-888U3-CS0-EXF). 2×2 binning was performed, and the luminescence intensities in 512×512 pixels were measured. 150 images were captured at a frame rate of 5.0 frame per second (fps) and an exposure time of 0.10 s. The luminescence intensities were then averaged for all pixels to calculate the average luminescence intensity within the vessel. This average intensity approximates the rate of sonochemical reactions within the entire vessel. Furthermore, the present study places significant emphasis on the decrease time of sonochemical reactions in quenching conditions. In this case, the luminescence intensities were normalized by the maximum values recorded during the designed recording time. This experiment was repeated for three times, and the quantitative value was averaged. The quantitative value was shown in Fig. 13.

### 2.2 Observation of bubbles visualized with a green laser

**Fig. 2:**
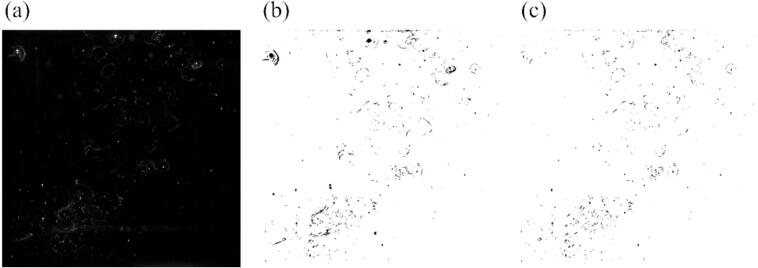
Photograph of bubbles visualized by the green sheet laser at 69 W: (a) the original image, (b) the binarized image of all bubbles and (c) the binarized image of small active bubbles. Bubbles smaller than 3000 μm^2^ were defined as small active bubble.

The presence of bubbles existing in the aqueous solution was determined through the use of a green laser sheet, which was irradiated with a 3 W continuous wave (CW) YAG laser at a wavelength of 532 nm. Subsequently, the scattered light from the bubbles were recorded by a high-speed camera (Photron, FASTCAM AX100). The frame rate was set at 125 fps, and the total number of recorded images was 5457. Fig. 2 indicates the photograph of bubbles, which is visualized by the laser sheet, with an ultrasonic power output of 69 W. These images were binarized using imageJ software. In this procedure, the original image shown in Fig. 2 (a) is converted into a binary image, as shown in Fig. 2 (b). The original photograph revealed the presence of two distinct phenomena: large bubbles trapped at the nodes and small active bubbles, a part of which forms streamers. It should be noted that streamers were identified as individual bubbles, although these bubbles appeared in a line [2], [45], [46] before the quenching event occurs. The separation of large inactive bubbles and small active bubbles was based on their size. Generally, the resonance bubble radius, *R*_0_ is used and the value is calculated by the following Minnaert equation.

$$\begin{array}{c} R_{0}=\frac{1}{\omega_{0}}\sqrt{\frac{3\gamma P_{0}}{\rho_{0}}} \end{array}$$

where *γ* is the specific heat ratio, *P*_0_ is the pressure amplitude, *ρ*_0_ is the liquid density. Under the experimental condition, the resonant radius is approximately 26 μm at most. In this study, the threshold of active or inactive bubbles is determined by the area of bubbles, that is, 3000 μm^2^. This value corresponds to approximately 60 μm in diameter, which is slightly larger than the resonant bubble diameter. The final binarized image of small active bubbles is shown in Fig. 2 (c). This experiment was repeated for three times to evaluate the reproducibility.

### 2.3 Flow velocity measured by Particle Image Velocimetry (PIV)

The flow velocity of acoustic streaming was measured by the PIV using the similar experimental equipment as section 2.2. The motion of fluorescent particles (EB-FLUOSTAR, KANOMAX) with the diameter of 15 μm were measured by the high-speed camera. The same green laser was used to excite the fluorescence in these particles, generating fluorescent light at a wavelength of 580 nm. To eliminate the scattered light from the impurity, cavitation bubbles and vessel wall, a high-pass filter was used. Only the particle motions were recorded by the same high-speed camera. The frame rate was 125 fps, and the number of captured images was 5457. Finally, the recorded images of particles were converted into flow velocity through a digital image correlation method. In the image analysis, the interrogation window size is 32 × 32 pixels, and an overlapping of window is 16 × 16 pixels. This experiment was repeated for three times to evaluate the reproducibility.

### 2.4 Calculation of decrease time

**Fig. 3:**
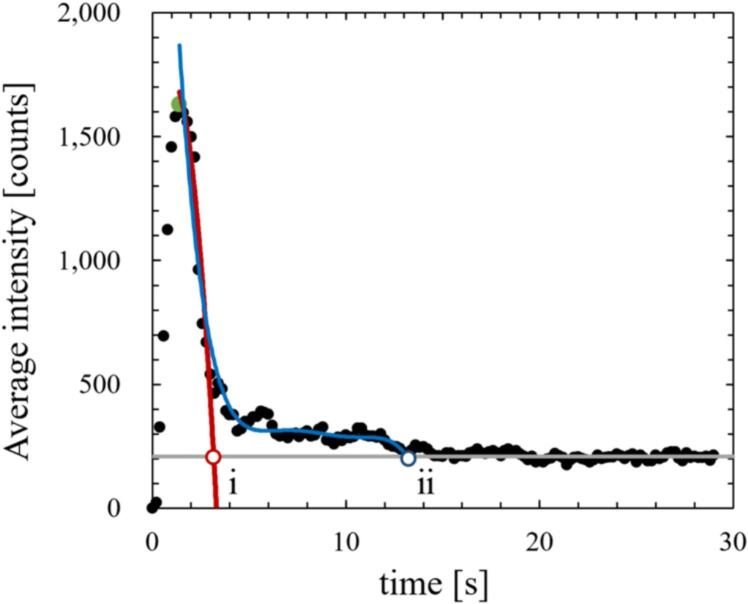
An example of average SCL intensity and calculation method of decrease time.

As discussed later in chapter 3, the SCL intensity, the number of formed bubbles, and the velocity of acoustic streaming decreased after a certain time passed from the ultrasonic irradiation in the quenching condition. To understand the relationship between the chemical reaction rate, the bubble formation rate and the velocity of acoustic streaming, we calculated the decrease time that it took for these physical quantities to decrease. However, the decrease time is difficult to define because these physical quantities do not decrease monotonically after quenching. Therefore, we calculated the decrease time of SCL, the area of all bubbles and the small active bubbles, and the flow velocity of acoustic streaming in two ways: i) the decrease time focusing on the initial decrease rate and ii) the time until the physical quantity completely decreases. Fig. 3 shows an example of the time variation of the average SCL. The SCL intensity increases rapidly, and decreases at approximately 3 seconds, and then, the decreasing rate of SCL intensity changed significantly at approximately 4 seconds. Finally, the SCL intensity decreases and plateaus at approximately 13 seconds. As shown in Fig. 3, the start time of the decrease was defined as the time when the value reached its maximum, and the decrease end time was defined as the time at which the measured value decreased to a stable value. When calculating the decrease time based on the initial decrease rate, we used an approximate polynomial curve, which is calculated using the physical quantities in the initial stage. Conversely, when calculating the time until the values completely decrease, the physical quantities were calculated based on the approximate polynomial curve, which is calculated using all the data before the quantities reached the plateau value.

## 3 Results and discussion

As explained in chapter 2, each experiment was repeated for three times. However, for the sake of clarity in results, we first show the date from a single experiment for Fig. 4, Fig. 5, Fig. 6, Fig. 7, Fig. 8, Fig. 9, Fig. 10, Fig. 11, Fig. 12. The reproducibility and quantitative evaluation were discussed in Fig. 13.

**Fig. 4:**
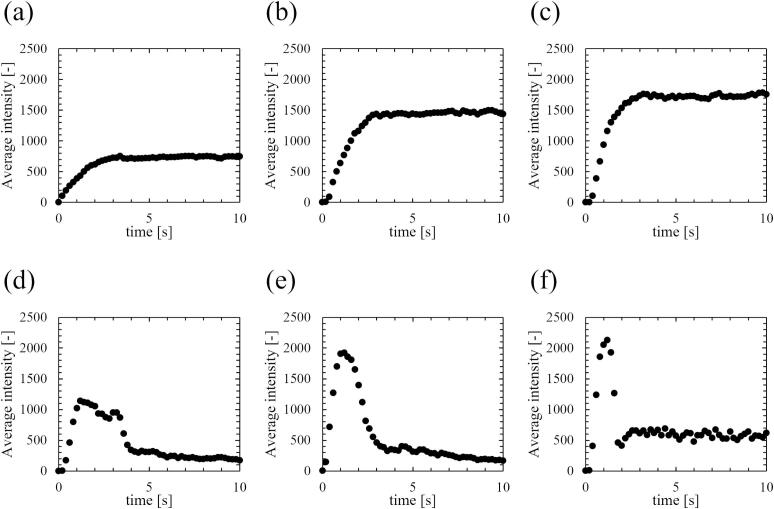
Time-dependent changes in averaged intensity of sonochemical luminescence. The ultrasonic power was (a) 18, (b) 33, (c) 43, (d) 69, (e) 84, and (f) 107 W.

**Fig. 5:**
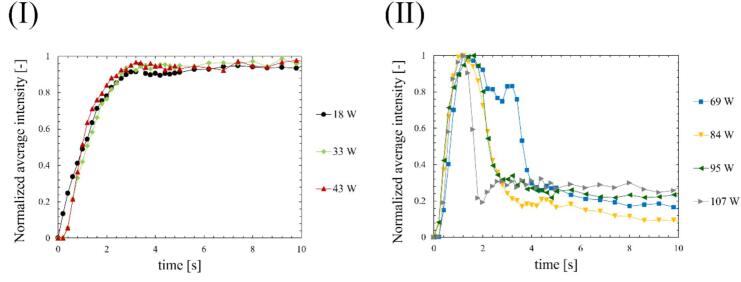
Time-dependent in normalized average intensity of sonochemical luminescence at the ultrasonic power of (Ⅰ) 18-43 W and (Ⅱ) 69-107 W.

**Fig. 6:**
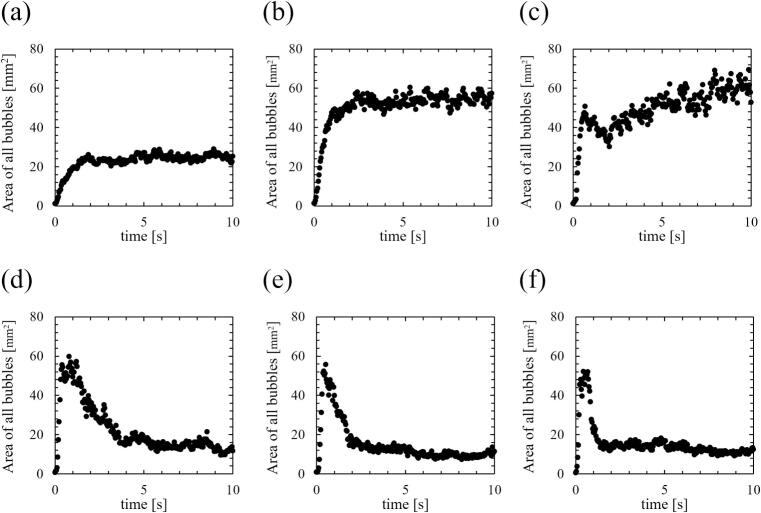
Time development of area of all bubbles. The ultrasonic power was (a) 18, (b) 33, (c) 43, (d) 69, (e) 84, and (f) 107 W.

**Fig. 7:**
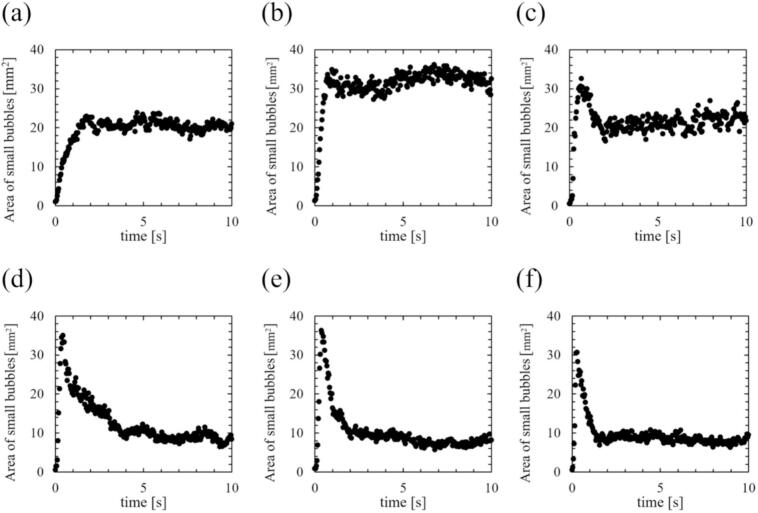
Time development of area of small active bubbles. The ultrasonic power was (a) 18, (b) 33, (c) 43, (d) 69, (e) 84, and (f) 107 W. The area values were calculated based on binarized photographs.

**Fig. 8:**
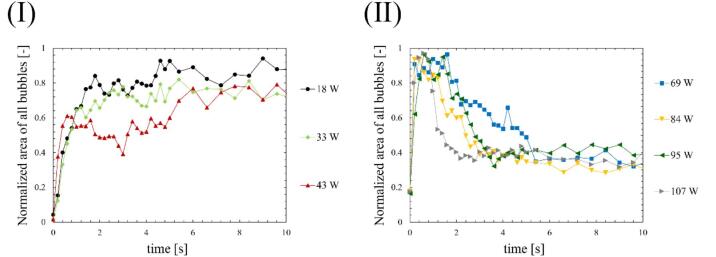
Time-dependent changes in normalized area of all bubbles at the ultrasonic power of (Ⅰ) 18-43 W and (Ⅱ) 69-107 W.

**Fig. 9:**
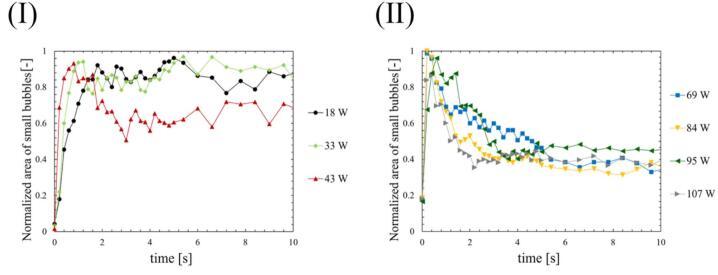
Time dependent changes in normalized area of small active bubbles at the ultrasonic power of (Ⅰ) 18-43 W and (Ⅱ) 69-107 W.

**Fig. 10:**
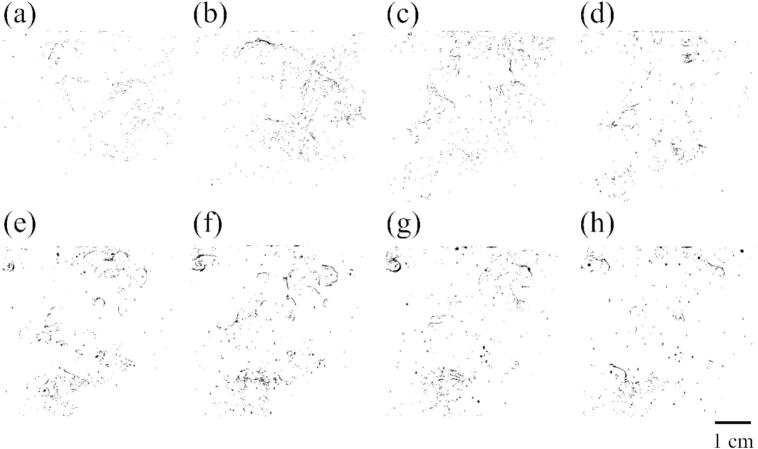
Binarized images of all bubbles with the ultrasonic power of 43 W at (a) 0.25, (b) 0.5, (c) 1.0, (d) 2.0, (e) 5.0, (f) 8.0, (g) 10, and (h) 15 s after ultrasonic irradiation starts.

**Fig. 11:**
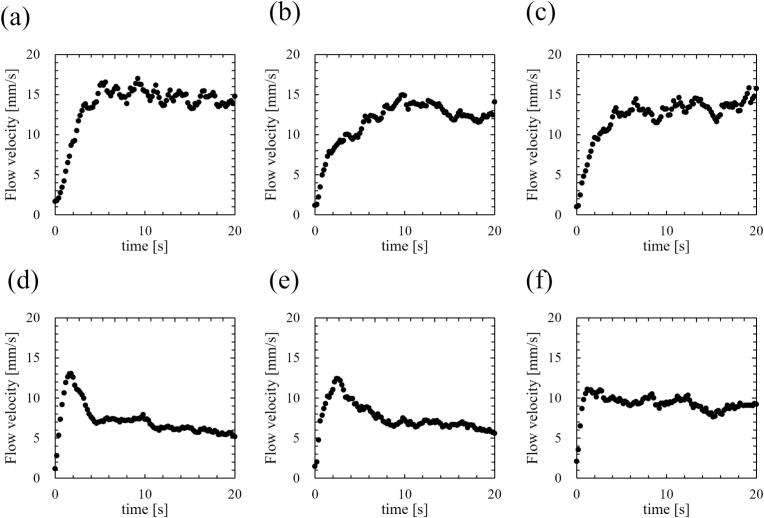
Time development of mean flow velocity of acoustic streaming at the ultrasonic power of (a) 18, (b) 33, (c) 43, (d) 69, (e) 84, and (f) 107 W.

**Fig. 12:**
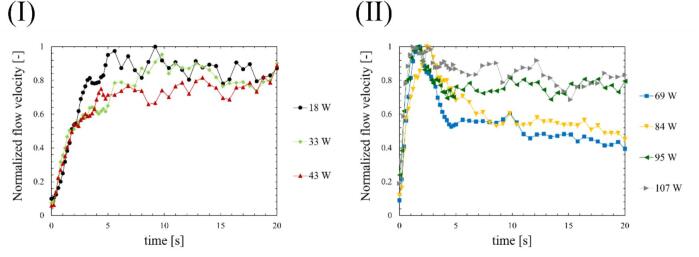
Time development of normalized mean flow velocity of acoustic streaming at the ultrasonic power of (Ⅰ) 18-43 W and (Ⅱ) 69-107 W.

**Fig. 13:**
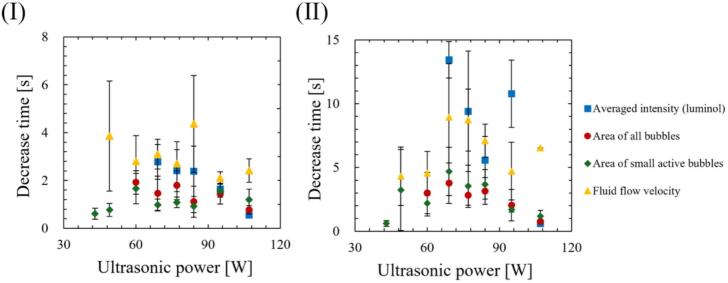
(Ⅰ) Decrease time of physical quantities focusing on the initial decrease rate, and (Ⅱ) the time until values completely decrease.

### 3.1 Sonochemical luminescence (SCL) observation

Fig. 4 shows the time-dependent changes in the average intensity of sonochemical luminescence at different ultrasonic powers. The intensities shown are the average luminescence intensity across the entire area. In Fig. 4, 0 seconds corresponds to the start time of ultrasonic irradiation. The results of the normalized luminescence intensity are shown in Fig. 5. At low power (a)–(c), the luminescence intensity increases to a certain value over time and then remains almost constant. On the other hand, at the ultrasonic powers above (d) 49 W, the luminescence intensity initially increased with irradiation time, though it decreased significantly after irradiation for a certain period. These results indicate that quenching occurs at medium and high ultrasonic powers. Sonochemical reactions have been reported to be correlated with bubble number and flow velocity of acoustic streaming [36]. Therefore, we carefully investigated the interaction between chemical reactions, the bubble number, and flow velocity immediately after ultrasonic irradiation. Furthermore, as shown in Fig. 5, the decrease time of luminescence intensity became shorter with increasing ultrasonic power. Thus, understanding the relationship between ultrasonic power and the decrease time of these reactions is important for comprehending the detailed process of quenching occurrence.

### 3.2 Observation of bubbles visualized by a green laser

The bubbles were visualized using a green sheet laser, and their area was calculated. The tendency of time variation of bubble number in a picture is almost the same as that of bubble area. Therefore, we used the bubble area to characterize the formed bubbles in the subsequent results. Fig. 6, Fig. 7 show the time-dependent changes in the areas of all bubbles and small active bubbles, respectively, with different ultrasonic powers. Fig. 8, Fig. 9 show the results of the normalized area of all bubbles and small active bubbles, respectively. As shown in Fig. 6, Fig. 7, Fig. 8, Fig. 9, at low power (a)–(c), the areas of both all bubbles and small active bubbles increased to a certain value and then remained constant. However, at an ultrasonic power of (c) 43 W, the area of all bubbles decreased once and then increased, while a slight decrease was observed in the area of small bubbles. At the power at which quenching occurred (d)–(f), the areas of all bubbles and small bubbles decreased after a period of irradiation. The decrease time was shortest at the high power of (f) 107 W. From these results, it can be concluded that the decrease in luminescence intensity is affected by the decrease in the area of small bubbles at the power where quenching occurs. Additionally, as the ultrasonic power increased, the increase rate in the bubble area became larger soon after the ultrasonic irradiation, as shown in Fig. 7, Fig. 8. Here, (c) 43 W is considered a transition region between the conditions before and after quenching.

To evaluate the transition region, we also carefully evaluated the increasing rate of the number of observed bubbles in a laser sheet. Fig. 10 shows the time development of all bubbles scattered by the laser sheet with an ultrasonic power of 43 W. Soon after the ultrasonic irradiation, the fine bubbles were formed, and the size of some of the bubbles gradually increases over time. Therefore, the reason why the increase in the area of all bubbles is thought to be due to an increase in the size of large bubbles trapped at the nodes.

### 3.3 Particle Image Velocimetry (PIV) measurement

Fig. 11 shows the temporal progression of the mean flow velocity of acoustic streaming, as measured by PIV, for different ultrasonic powers. Similar to the SCL intensity, the magnitude of flow velocity is averaged across the entire domain. Ultrasound was irradiated at 0 second in Fig. 11, and the mean flow velocity was approximately 2 mm/s under fully developed conditions. Fig. 12 shows the mean flow velocity normalized by the maximum value. As can be seen, at low power (a)–(c), the mean flow velocity of acoustic streaming increased to a certain degree after irradiation and remained relatively constant. However, these values were fluctuating due to the turbulent velocity fluctuations. On the other hand, at ultrasonic powers where quenching occurred (d)–(e), with the exception of (f), the mean flow velocity initially increased, followed by a subsequent decrease after irradiation for a certain period. In the case of high ultrasonic power (f), the decrease in flow velocity was extremely small after the flow velocity becomes the maximum. It is to be noted that as the ultrasonic power increased, the increase rate of the mean flow velocity of acoustic streaming became larger soon after the ultrasonic irradiation in the same way as SCL intensity.

In the subsequent section, the mechanism of quenching occurrence will be discussed, along with the relationship between SCL intensity, the number of formed bubbles, and the mean fluid velocity of acoustic streaming. These relationships will be discussed based on the measured experimental results.

### 3.4 Discussion

Firstly, we discuss the relationship between SCL, the number of formed bubbles and fluid velocity of acoustic streaming at the quenching power. Fig. 13 shows the decrease times of the SCL intensity, the area of bubbles, and the flow velocity of acoustic streaming based on the results above, which were calculated in two ways: (Ⅰ) decrease time focusing on the initial decrease rate, and (Ⅱ) the time until physical quantities completely decrease as explained in section 2.4. As shown in Fig. 13, the area of bubbles exhibited the most rapid decrease among the three factors. Consequently, the predominant factor contributing to the quenching process is the number of formed bubbles. The suppression of formed bubble is thought to be caused by sound pressure deterioration of ultrasound wave superposition, as found in our previous study [36]. The sound pressure amplitude increases with increasing ultrasonic power, intensifying the generation of sound waves by the cavitation bubble oscillations. As a result, the superposition of ultrasonic waves is deteriorated, resulting in nonlinear ultrasonic waves that diminish the amplitude of bubble oscillations, rectified diffusion and bubble growth. A secondary effect of this phenomenon is the gradual collapse of the bubbles, which is caused by the reduced bubble expansion. Subsequent to the decrease in the number of formed bubbles, a corresponding decrease in sonochemical luminescence intensity was observed. This phenomenon is thought to result from the suppression of small active bubbles that contribute to the sonochemical reactions. Furthermore, it is considered that even after bubble oscillations have been weakened, oxidants such as H_2_O_2_ generated from OH radicals, persist in the solutions. These oxidants have the capacity to react with luminol in the solution in close temporal proximity to the suppression of active bubbles. Consequently, the occurrence of partial chemical reactions persists, leading to an extended decrease time as depicted in Fig. 13 (Ⅱ). Lastly, the flow velocity of acoustic streaming decreased at the quenching condition because the inertia of the fluid flow of acoustic streaming is large, and it takes long time for viscous dissipation. Based on these discussions, we can explain the reason why the decrease in flow velocity soon after the ultrasonic irradiation is minimized at high power (f) 107 W. The acoustic streaming that occurs in the kHz ultrasonic range, under acoustic cavitation condition, is mainly driven by the attenuation of sound waves due to bubbles oscillations. Therefore, the flow velocity is found to be correlated with the number of formed bubbles. At high power (f) 107 W, the number of formed bubbles decreases so rapidly as seen in Fig. 6, Fig. 7, Fig. 8, Fig. 9, and it is thought that the number of formed bubbles decreases while the fluids are accelerating. Consequently, the flow velocity does not decrease in the initial period of ultrasound irradiation.

Secondly, the mechanism of quenching occurrence is discussed. Previous studies have reported that harmonic-induced waveform distortion suppresses bubble oscillations due to reduced rectified diffusion, which leads to decrease in bubble generation, chemical reactions, and flow velocity of acoustic streaming [36]. The present study discovered the direct evidence indicating that the decrease in the bubble generation is associated with a decrease in chemical reactions and flow velocity of acoustic streaming. Consequently, these findings serve to complement the mechanism proposed in our previous study [36].

**Fig. 14:**
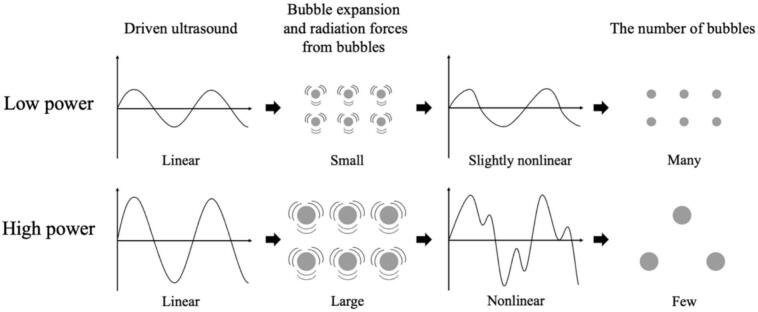
Phenomenon occurring in the early stage of ultrasonic irradiation at low- and high-power conditions.

Moreover, an additional mechanism can be interpreted from the aforementioned results. As the ultrasonic power increases, the time required for quenching decreases, as shown in Fig. 13. This result indicates that quenching occurs more rapidly under conditions of high sound pressure amplitude. Considering that harmonic waves are induced by the sound radiation from bubbles, this suggests that as the sound pressure amplitude increases, the sound radiation from bubbles becomes more significant. The factors contributing to this enhanced acoustic emission from the bubbles are considered to be equilibrium bubble radius or the number of formed bubbles. As demonstrated in Fig. 6, Fig. 7, Fig. 8, Fig. 9, the increase rate of the bubble area becomes larger with increasing the ultrasonic power, indicating that the bubble growth rate due to rectified diffusion increased with increasing the ultrasonic power. Fig. 14 illustrates the phenomenon occurring in the early stage of ultrasonic irradiation schematically. At low ultrasonic power, the bubble growth due to rectified diffusion becomes small. Consequently, the sound radiation from the oscillating bubbles becomes small, and the ultrasonic waveform remains largely undistorted. On the other hand, at high power, the bubble growth rate due to rectified diffusion increases. Therefore, as sound radiation and absorption by the bubbles increase, the sound waves are distorted significantly. This distortion reduces the rectified diffusion, and bubble generation is suppressed [36]. These factors cause quenching of sonochemical reactions. Therefore, the primary reason for the reduction in the decrease time of physical quantities with increasing the ultrasonic power is considered to be mainly due to the rectified diffusion, which is varied due to the ultrasonic wave distortion.

This study provides recommendations for practical applications. Since we have determined the approximate reduction time for sonochemical reactions, it is preferable to apply ultrasound intermittently at intervals corresponding to that time. Previous reports have shown that intermittent ultrasound irradiation improves its efficiency [47], and our results support the findings observed in the previous study. The results shown in this study also suggest that even more efficient operations are possible because the decrease time of sonochemical reactivity decreases with increase of ultrasonic power.

## 4 Conclusions

In this study, sonochemical reactions immediately after ultrasonic irradiation were investigated to understand the occurrence process of power-induced quenching. The interaction between the number of formed bubbles, sonochemical reaction rate and flow velocity of acoustic streaming was investigated through the direct observation of bubbles using laser sheet, SCL intensity, and PIV measurement. The findings in this study are summarized below:

• At the low ultrasonic power, the sonochemical luminescence intensity increased to a certain value with irradiation time and then maintained the intensity after the sufficient acoustic bubbles were generated.
• At the ultrasonic power where the power-induced quenching occurred, the sonochemical luminescence intensity decreased after irradiation for a certain period. First, the number of formed bubble decreases due to the waveform distortion caused by sound waves radiated by bubbles’ oscillations. After the bubble number decreased, the SCL intensity and the flow velocity of acoustic streaming decreased.
• As the ultrasonic power increases, the decrease time of SCL intensity, number of formed bubbles, and the fluid flow velocity of acoustic streaming becomes shorter, and these phenomena is interpreted by rectified diffusion, the mass flux of which is largely varied due to the ultrasonic wave distortion.
• At extremely high ultrasonic power, although the chemical reactions and bubble number decreased after irradiation for a certain period, the flow velocity did not decrease because the bubble number decreased so rapidly and the fluid has insufficient time to be accelerated.

## CRediT authorship contribution statement

**Ryota Aoki:** Writing – original draft, Visualization, Investigation, Formal analysis, Data curation. **Takuya Yamamoto:** Writing – review & editing, Supervision, Project administration, Funding acquisition, Conceptualization.

## Declaration of competing interest

The authors declare that they have no known competing financial interests or personal relationships that could have appeared to influence the work reported in this paper.
